# Cross-Sectional and Longitudinal Effects of *CREB1* Genotypes on Individual Differences in Memory and Executive Function: Findings from the BLSA

**DOI:** 10.3389/fnagi.2017.00142

**Published:** 2017-05-16

**Authors:** Claudia Wolf, Yang An, Toshiko Tanaka, Murat Bilgel, Christopher Gonzalez, Melissa Kitner Triolo, Susan Resnick

**Affiliations:** ^1^Laboratory of Behavioral Neuroscience, National Institute on Aging, National Institutes of Health, BaltimoreMD, United States; ^2^Psychological Research Methods, Department of Psychology, Humboldt University BerlinBerlin, Germany; ^3^Translational Gerontology Branch, National Institute on Aging, National Institutes of Health, BaltimoreMD, United States; ^4^Clinical Research Branch, Medstar Health Research Institute, BaltimoreMD, United States; ^5^Multimodal Imaging Laboratory, Department of Neurosciences, University of California San Diego, La JollaCA, United States

**Keywords:** cognitive aging, memory, executive function, *CREB1* genotypes, longitudinal study

## Abstract

**Purpose:** Previously, we have shown that the SNP rs10932201 genotype of the cyclic AMP responsive element binding protein 1 gene (*CREB1*) contributes to individual differences in executive and memory function at the neural system and behavioral levels in healthy, young adults. However, longitudinal effects of *CREB1* genotypes on cognition have not yet been addressed. Furthermore we were interested in replicating associations between *CREB1* genotypes and human cognition in previous cross-sectional studies and explore whether APOE𝜀4 status might modify these relations.

**Materials and Methods:** We investigated whether common, independent tag SNPs within *CREB1* (rs2253206, rs10932201, rs6785) influence individual differences in age-related longitudinal change and level of executive function and memory performance independent of baseline age, sex, APOE𝜀4 status, and education. Our analysis included data from cognitively unimpaired older adults participating in the Baltimore Longitudinal Study of Aging. Eleven measures from six cognitive tests (sample sizes range 617–786) were analyzed using linear mixed effects and generalized estimating equations models. Mean baseline age ranged from 50 to 69 years and mean time of follow-up (interval) ranged from 8 to 22 years.

**Results:** We found significant effects of all three *CREB1* SNPs on performance level and/or longitudinal change in performance based on eight measures assessing semantic memory, episodic memory, or both executive function and semantic memory. SNP rs10932201 showed the most significant and largest effect (Cohen’s *d* = -0.70, *p* < 0.01) on age-related longitudinal decline of semantic memory. Additionally, we show interactions between all three *CREB1* SNPs and APOE𝜀4 status on age-related longitudinal declines and levels of memory and executive function.

**Conclusion:** Our results suggest that *CREB1* genotypes independently and by interactions with APOE𝜀4 status contribute to individual differences in cognitive aging.

## Introduction

Human cognitive aging is highly variable across individuals (Lindenberger, 2014). Identifying genetic factors that contribute to interindividual variability in performance and age-related decline of specific cognitive functions can inform us about the underlying neuromolecular networks. Genetic variations in the *CREB1* gene (HGNC: 2345) are of interest because CREB signaling pathways, by regulating gene expression, contribute to the adaptation of neuronal properties in brain regions important for memory and executive functions (Benito and Barco, 2010; Rapanelli et al., 2010; Pozzi et al., 2011; Sanchez-Huertas and Rico, 2011; Leckie et al., 2014). Memory and executive function are susceptible to age-related decline and early neurodegeneration (Ronnlund et al., 2005; Grober et al., 2008; Goh et al., 2012; Weintraub et al., 2012). Age-related changes in CREB signaling pathways have been reported in animal models, especially in relation to deficits in long-term and working memory, and brain regions such as the hippocampus and prelimbic prefrontal cortex (Brightwell et al., 2004; Kudo et al., 2005; Monti et al., 2005; Vandesquille et al., 2013). In young and aged adult mice, following inhibition the reactivation of CREB signaling pathways in the hippocampus has been consistently shown to increase CREB-dependent transcription and rescue deficient neuroplasticity and memory (Genoux et al., 2002; Smith et al., 2009; Yiu et al., 2011; Adlard et al., 2014). Similarly the aging-related decrease in long-term memory found in the control rats could be prevented by CREB gene transfer in the hippocampus (Mouravlev et al., 2006). Recent evidence also supports CREB activation levels as a potential biomarker for cognitive decline in Alzheimer’s disease (Bartolotti et al., 2016).

The APOE𝜀4 genotype has been related to lower memory and greater decline in cognitive performance or memory in cognitively unimpaired participants (Yaffe et al., 1997; Bretsky et al., 2003; Caselli et al., 2004, 2009, 2011; Packard et al., 2007; Beydoun et al., 2012; Schiepers et al., 2012; Samieri et al., 2014), which could be dependent on CREB signaling pathways (Chen et al., 2010; Qiao et al., 2014; Liu et al., 2015). For example, contrary to APOE3, APOE4 stimulates the transcriptional activity of CREB by activating the extracellular signal-regulated kinase (ERK) pathway in hippocampus neurons (Ohkubo et al., 2001). Accordingly, CREB may be important in elucidating pathways related to individual differences in cognitive decline.

Previously we reported cross-sectional effects of the *CREB1* SNP rs10932201 genotype on executive and memory function at the neural system and behavioral levels in healthy, young adults (Wolf et al., 2015). Longitudinal effects of *CREB1* genotypes on cognition have not yet been addressed. Here, we investigate whether common variants within the *CREB1* gene, after adjustment for baseline age, sex, APOE𝜀4 status, and education, influence the performance level and longitudinal change in performance of executive and memory function during aging. Furthermore we address whether APOE𝜀4 status and *CREB1* genotypes interactively influence cognitive aging.

## Materials and Methods

### Participants

Participants of the Baltimore Longitudinal Study of Aging (BLSA) are community-dwelling volunteers. Continuous recruitment into the BLSA has occurred since 1958. Exclusion criteria at enrollment were past or present psychiatric, neurological, cardiovascular, severe gastrointestinal, kidney or liver disease, diabetes, birth defects, established genetic diseases, inability to perform daily self-care without assistance, inability to walk independently for at least 400 m without assistance and without developing symptoms, inability to perform normal activities of daily living without shortness of breath, active cancer (any activity in the last 10 years), except for locally limited basal cell cancer, clinically significant hormonal dysfunction, muscle-skeletal conditions due to diseases or traumas, any long-term medical treatment, important sensory deficits, having severe English language difficulties, physical illness and weight over 136 kg. Specifically for our analysis we restricted the sample to Caucasian Americans with data available for the *CREB1* SNPs of interest, APOE𝜀4 genotype status and education. Cognitive status of participants was assessed at every visit. Clinical consensus case conferences were performed if the Blessed Information Memory Concentration score was ≥4 (Fuld, 1978), the Clinical Dementia Rating score was ≥0.5 (Morris, 1993), or concerns were raised about a participant’s cognitive status, and upon death in case participants were in the autopsy study. Based on clinical and neuropsychological data, cognitive impairment and timing of onset were determined. Data after the onset of cognitive impairment for individuals who subsequently developed impairment were excluded from the current analyses.

The number of participants, time of follow-up and demographics varied depending on the cognitive measure (Supplementary Table S1). This study was carried out in accordance with the recommendations of NIH guidelines, the institutional review board National Institute of Environmental Health Sciences with written informed consent from all subjects. All subjects gave written informed consent in accordance with the Declaration of Helsinki at each study visit. The protocol was approved by the institutional review board National Institute of Environmental Health Sciences.

### Acquisition of Cognitive Data

Data were collected for comprehensive medical, physiological, and neuropsychological evaluations approximately every 2 years and since 2000 every year for participants older than 80 years. We selected six cognitive tests that have previously been shown to be sensitive to individual differences in primarily memory (Golski et al., 1998; Balthazar et al., 2008; Goh et al., 2012) or both memory and executive function (Paula et al., 2013; Shao et al., 2014) during healthy cognitive aging. These tests included the Boston Naming Test (BNT), the Benton Visual Retention Test (BVRT), California Verbal Learning Test (CVLT), the Clock Drawing Test (CLOCKs), the Category and Letter Fluency Tests. The data used in our analysis was acquired for the BVRT between 1960 and 2014, for the CVLT between 1993 and 2014, and for the remaining three tests between 1984 and 2014. Until 2005 the CLOCKs and Fluency Tests were only obtained from participants with age ≥60 years instead of at every visit. The specific measures used from each cognitive test, the range for each measure and a summary of the cognitive processes contributing to each measure are shown in Supplementary Table S2.

### Detailed Description of Cognitive Tests

The BVRT measures visual constructional skill and short-term figural memory. Participants study 10 line drawings including one to three geometric figures for 10 s each, and then immediately reproduce them from memory using pencil and paper. The designs become more difficult over the 10 trials. The BVRT has been administered to BLSA participants since 1960. The dependent variable was the total number of errors, which were scored independently by two trained technicians, employing a modified error scoring method, based on the method provided in the BVRT manual (Benton, 1974).

The BNT (Kaplan et al., 1983) is a measure of object recognition and semantic retrieval. Participants identify and name a series of line drawings of objects, beginning with common objects and ending with infrequent objects. Administration in the BLSA involves cueing incorrectly named objects, with either a stimulus cue for perceptual errors or a phonemic cue for semantic errors. This test has been administered to BLSA participants 70 years and older since 1986 and to BLSA participants 60 years and older since 1990. The dependent measure was the number of words out of 60 correctly named without cues.

The CVLT assesses verbal learning and memory (Delis et al., 1987). Five learning trials of 16 shopping items, with four items from each of four semantic categories are presented orally and the sum of the five trials provides a measure of immediate free recall. In addition, short- and long-delayed free recall, short- and long-delayed cued recall, and recognition memory are assessed. The CVLT has been administered to BLSA participants since 1993. Four dependent measures were used in the present analyses: total number of items recalled across the five immediate recall learning trials, short-delay free recall, long-delay free recall, and recognition memory with maximum scores of 80, 16, 16, and 16, respectively.

The Clock Drawing Test (CLOCK) is a test of constructional and visuospatial ability (Rouleau et al., 1992). Three clocks were drawn; two from memory (3:25, 11:10) and one was copied (11:10). Participants were asked to draw a clock, put in all of the numbers and set the hands to the specified time. The Clock Drawing task has been administered to BLSA participants 70 years and older since 1986 and to BLSA participants 60 years and older since 1990. The clock face was given up to 2 points, and the numbers and hands are given up to 4 points, for a total of 10 points.

Letter and category fluency (Fluencies) tests are measures of fluent language production and executive function. Participants were given 60 s to generate as many words as possible beginning with specific letters (F, A, S) (Benton, 1968) and from specific categories (fruits, animals, vegetables) (Newcombe, 1969). Fluency measures have been administered to BLSA participants 70 years and older since 1986, to BLSA participants 60 years and older since 1990 and to all participants since 2005. The total numbers of correct words generated in 60 s, across the three trials of letter and category fluency, were the dependent measures of interest.

### Measuring Symptoms of Depression

We used the Center for Epidemiological Studies Depression (CESD) scale (Radloff, 1977) to screen for symptoms of depression. Based on our largest sample (*N* = 786), this score was unavailable for 50 participants (6.4%) partly due to the fact that the CESD scale was unavailable before 1977.

### Genotyping

In BLSA, blood samples were collected for DNA extraction, and genome-wide genotyping was completed for 1231 subjects using Illumina 550K. Genotype data were available from 848 participants of European ancestry using a call rate of >98.5% without sex discrepancy based on homozygosity rates. 501,704 autosomal SNPs passed quality control (completeness > 99%, MAF > 1%, HWE > 10-4) were used for imputation (Price et al., 2006). Imputation of ∼2.5 million HapMap SNPs were imputed using CEU sample (Phase II, release 22, build 36) as a reference using MACH (Tanaka et al., 2009). Based on genotyping and imputing, genotype data for eight SNPs within the *CREB1* gene (Supplementary Table S3a) were available for those participants with cognitive data.

APOE𝜀4 genotype status was determined by polymerase chain reaction amplification of leukocyte DNA followed by HhaI digestion and product characterization (Hixson and Vernier, 1990) and by the TaqMan assay systems based on several single nucleotide polymorphisms located in the ApoE gene (Koch et al., 2002).

### Statistical Analysis of Genetic and Cognitive Data

We used the SPSS (IBM SPSS Statistics 23) and R (version 3.2.2) for Windows for our statistical analysis of genetic and behavioral data.

Bivariate Pearson’s correlation coefficients (two-tailed) were used to test pair-wise LD between SNPs. The cutoff for LD was set to an *R*^2^ value of 0.6. Chi-squared tests were used to check HWE [α-level 0.05; 2 degrees of freedom (df)] and differences in genotype groups between sex (α-level 0.05; 2 df). ANOVAs were used to analyze differences in age, education (α-level 0.05; two-tailed; 783 df) and score for symptoms of depression (733 df) between SNP-based genotype groups. Only SNPs rs2253206, rs10932201, and rs6785 identified as independent were further analyzed because they were not in high LD defined as an *R*^2^ of ≥0.6.

### Calculation of Cognitive Performance Measures

Performance measures were based on the total sum of correct responses for the BNT, CLOCKs, CVLT free recall measures, Category and Letter Fluency and the total sum of errors for the BVRT. The CVLT-recognition correct was the sum of all correctly recognized targets (total hits). The CVLT-recognition discriminability is another performance accuracy measure, which was calculated as *Z*-score for the probability of hit [Z(p(hit))] minus Zscore for the probability of false alarm/incorrectly recognized as target [Z(p(FA))]. The CVLT-recognition response bias measures the degree of performance bias, which was calculated as *Z*-score for the probability of total hits plus *Z*-score for the probability of false alarms [-0.5^∗^Z(p(hit)) + Z(p(FA))]. Values closer to zero indicate less biased performance for the CVLT-recognition response bias measure, a value of zero indicates a completely unbiased or neutral response. Because of missing information on FA, the CVLT-recognition response bias and discriminability measures could not be calculated for one visit of a subject with multiple visits. Scores with zero total hits were excluded for nine visits from subjects with more than one visit and one subject with a single visit.

These 11 cognitive measures were used as dependent variables in our models. Baseline age, education, and time of follow-up (interval) were continuous predictors, and sex, APOE𝜀4 status, and SNP were nominal predictors. There were 15 APOE𝜀4/4 carriers in our largest sample, which unfortunately is insufficient to carry out a subgroup specific analysis. Therefore APOE𝜀4 status was defined as APOE𝜀4 positive for participants with at least one APOE𝜀4 allele, while other participants were defined as APOE𝜀4 negative. For the *CREB1* SNPs, the genotype group homozygous for the global major allele was used as the reference genotype group (rs2253206 → AA, rs10932201 → GG, and rs6785 → GG). We modeled the genotype effect using either all three genotype groups or two groups by combining heterozygous and homozygous genotype groups for the global minor allele versus the genotype group homozygous for the global major allele (reference group).

We used generalized estimating equations (GEE) models with Poisson distribution for CLOCK based measures after their transformation (maximal possible score minus observed score) to model the counts. Accordingly, originally high performance scores were turned into low performance scores. These transformed scores were back-transformed for the graphical presentations. Linear mixed effects models (LME) with [unstructured covariance for the random effects] were used for all other cognitive measures. We included as fixed effects baseline age, sex, APOE𝜀4 status and education, time of follow-up (interval), SNP and interactions of interest: baseline age × interval, sex × interval, APOE𝜀4 status × interval, and SNP × interval, into both the LME and GEE models. Intercept and interval for each subject were also included as random effects with the unstructured covariance in the LME models, and exchangeable covariance was used for repeated measures in the GEE models. We mean centered baseline age, sex, APOE𝜀4 status and education for simultaneous interpretation of main effects and interactions in the models. The main effect of interval is the slope for the mean of baseline age, sex, APOE𝜀4 status, and education for the SNP genotype reference group. Using these models allowed us to analyze cross-sectional effects (baseline age, sex, APOE𝜀4 status, education and each SNP) as well as interactions of interest with the longitudinal effect of interval simultaneously.

We used Cohen’s d effect size definition implemented in LME models, where the group difference is estimated based on the beta coefficient from LME regression models and standard deviations of the data are estimated from the covariance structure of the random effects. For the GEE models, Cohen’s d effect size was calculated by using the estimates of variance-covariance matrix from the random effects in generalized linear mixed models.

We also tested for putative interactions between the *CREB1* SNPs and APOE𝜀4 status by adding to all our models the interaction between SNP and APOE𝜀4 status as well as the interaction between SNP, APOE𝜀4 status and interval. For those models showing significant interactions, we used *post hoc* test to identify group differences that underlie the overall effect.

## Results

### *CREB1* Genotypes

Allele frequencies for eight SNPs within the *CREB1* gene are shown in Supplementary Table S3a. Based on MAF < 0.05 in our largest sample (*N* = 786) SNP rs16839883 (MAF = 0.01) and rs2709393 (MAF = 0.02) were excluded from further analysis. Results of pairwise linkage disequilibrium analysis (Supplementary Table S3b) revealed strong linkage between rs2253206 and rs2254137 (*R*^2^ = 0.6) as well as rs2551928, rs1045780, and rs6785. Therefore we selected rs2253206, rs10932201, and rs6785 (range of *R*^2^ = 0.1–0.2) for further analysis as independent tag SNPs. None of the *CREB1* SNPs were in linkage disequilibrium with APOE𝜀4 genotype status (all *R*^2^ = 0). All three independent SNP genotypes were distributed according to HWE (Supplementary Table S3c) and were not significantly different by sex, baseline age, education or symptoms of depression score (Supplementary Table S3d).

### Sensitivity of Cognitive Measures to Aging and Baseline Age

We observed significant longitudinal declines (effect of interval) in performance for all our measures of memory and executive function as well as lower cross-sectional performance levels and greater longitudinal declines in performance with increased baseline age for most of these measures (Supplementary Table S4).

### Cross-Sectional and Longitudinal Effects of *CREB1* Genotypes on Individual Differences in Memory and Executive Function

**FIGURE 1:**
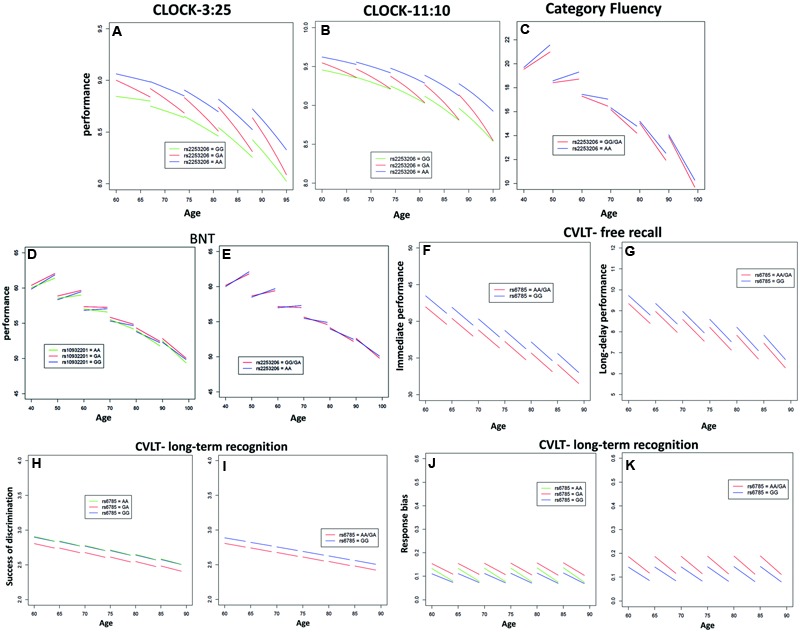
**Significant effects of *CREB1* genotypes on the longitudinal population-average trajectories for performance measures of executive function and/or memory (A)** CLOCK-3:25, **(B)** CLOCK-11:10, **(C)** Category Fluency, **(D,E)** BNT, **(F)** California Verbal Learning Test (CVLT)-immediate free recall, **(G)** CVLT-long-delay free recall, **(H,I)** CVLT-recognition discriminability, and **(J,K)** CVLT-recognition response bias based on the predicted values after adjusting for baseline age, sex, education, and APOE𝜀4 status. All the effects were estimated using linear mixed effects (LME) models except for CLOCK measures where generalized estimating equations (GEE) models were used.

Performance of executive function and semantic memory was significantly affected by SNP rs2253206, showing cross-sectional effects for both CLOCK-based measures and a longitudinal effect for the CLOCK-11:10-based measure (**Figures 1A,B** and Supplementary Tables S5, S6). The AA genotype was associated with beneficial effects on both performance level and change in executive function and semantic memory, while the GG genotype was associated with lower performance level and the GA genotype with greater decline in performance. Significantly less longitudinal decline in performance of executive function and semantic memory measured by Category Fluency was observed in the genotype group AA compared to the group with GG/GA genotype for SNP rs2553206 (**Figure 1C** and Supplementary Table S7). None of the SNPs showed a significant effect on letter fluency (Supplementary Table S8). In addition to the significant effects of SNP rs2253206, at trend level SNP rs6785 also affected performance level and SNP rs10932201 influenced the decline in executive function and semantic memory (Supplementary Table S5).

Genotype groups AA or GA compared to GG for SNP rs10932201 (**Figure 1D** and Supplementary Table S9) and GG/GA compared to AA for SNP rs2253206 (**Figure 1E** and Supplementary Table S9) showed significantly greater longitudinal decline in performance of semantic memory and language measured by BNT.

The performance level of verbal episodic memory during immediate as well as delayed free recall measured by CVLT was significantly lower in genotype group AA/GA compared to GG for SNP rs6785 (**Figures 1F,G** and Supplementary Tables S11, S12). We found also a significant main effect of this SNP on the performance of episodic long-term recognition memory measured by CVLT-recognition discriminability, which reflects the success of discriminating target from non-target items (**Figures 1H,I** and Supplementary Table S14). Again, group AA/GA compared to GG performed at a lower level for this cognitive measure. The performance level of episodic long-term recognition memory measured by CVLT-recognition response bias was significantly lower indicated by greater response bias (further away from zero) in genotype group AA/GA compared to GG for SNP rs6785 (**Figures 1J,K** and Supplementary Table S15). At trend level cross-sectional performance of episodic short-term memory was also influenced by SNP rs6785 (Supplementary Table S13). Trends for cross-sectional and longitudinal effects of SNP rs10932201 were also observed on visual immediate episodic memory (Supplementary Table S10). SNP rs2253206 showed an effect on the performance level of verbal immediate episodic memory at trend level (Supplementary Table S11). Please see **Table 1** for an overview of *CREB1* SNPs significantly affecting cognitive measures.

**Table 1:** Cross-sectional and longitudinal effects of *CREB1* genotypes on cognitive measures controlling for baseline age, sex, APOE𝜀4 status, and education.

Cognitive functions	Cognitive measure	SNP and grouping (group size)	Estimate SNP main effect (Cohen’s d)	*p*-value SNP main effect	Estimate interaction SNPx interval (Cohen’s d)	*p*-value interaction SNPx interval
Executive function and semantic memory	CLOCK-3:25 trans-formed	rs2253206 overall		**0.018**^∗^		0.053
		GG **(193)** vs. AA **(129)**	0.209 (0.50)	**0.015**^∗^	-0.006 (-0.88)	0.569
		GG vs. GA **(312)**	-0.145 (-0.35)	**0.022**^∗^	0.016 (0.36)	**0.016**^∗^
		GA vs. AA	0.064 (0.15)	0.457	0.010 (1.47)	0.334
	CLOCK-11:10 trans-formed	rs2253206 overall		**0.031**^∗^		**0.029**^∗^
		GG vs. AA	0.364 (0.36)	**0.013**^∗^	-0.008 (-0.23)	0.529
		GG vs. GA	-0.182 (-0.18)	0.089	0.025 (0.72)	**0.010**^∗^
		GA vs AA	0.182 (0.18)	0.222	0.017 (0.49)	0.147
	Category Fluency	rs2253206	-0.146 (-0.05)	0.583	-0.050 (-0.35)	**0.025**^∗^
		GG/GA **(*N* = 575)** vs AA **(150)**				
Semantic memory and language	BNT	rs10932201 overall		0.226		**0.004**^∗∗^
		AA **(138)** vs. GG **(180)**	0.191 (0.05)	0.672	-0.069 (-0.70)	**0.013**^∗^
		AA vs. GA **(303)**	-0.353 (-0.10)	0.322	0.003 (0.03)	0.894
		GA vs. GG	0.544 (0.15)	0.096	-0.072 (-0.70)	**0.002**^∗∗^
		rs10932201	0.434 (0.12)	0.158	-0.071 (-0.70)	**0.001**^∗∗^
		AA/GA vs. GG				
		rs2253206	0.172 (0.05)	0.346	-0.054 (-0.50)	**0.039**^∗^
		GG/GA **(494)** vs. AA **(127)**				
Episodic memory	CVLT-immediate free recall	rs6785	-1.512 (-0.16)	**0.021**^∗^	0.003 (-0.01)	0.960
		AA/GA **(265)** vs. GG (**468)**				
	CVLT-long-delay free recall	rs6785	-0.384 (-0.15)	**0.045**^∗^	-0.004 (-0.03)	0.809
		AA/GA vs. GG				
	CVLT-recognition discriminability	rs6785 overall		**0.038**^∗^		0.872
		AA **(32)** vs. GG **(468)**	0.020 (0.05)	0.825	-0.004 (-0.21)	0.601
		AA vs. GA **(233)**	0.116 (0.26)	0.206	-0.004 (-0.21)	0.624
		GA vs. GG	-0.961 (-0.22)	**0.013**^∗^	-0.000 (-0.01)	0.967
		rs6785	-0.082 (-0.18)	**0.026**^∗^	-0.006 (-0.32)	0.862
		AA/GA vs. GG				
	CVLT-recognition response bias	rs6785 overall		**0.021**^∗^		0.182
		AA vs. GG	0.013 (0.08)	0.743	-0.003 (-0.30)	0.480
		AA vs. GA	-0.034 (-0.22)	0.393	0.000 (0.02)	0.967
		GA vs. GG	0.047 (0.30)	**0.005**^∗∗^	-0.003 (-0.30)	0.074
		rs6785	0.042 (0.27)	**0.008**^∗∗^	-0.003 (-0.30)	0.064
		AA/GA vs. GG				

*The reference genotype group is underlined. Significant effects are shown in bold with ^∗^ indicating significance at *p* = 0.05 and ^∗∗^ at *p* = 0.01.*

### APOE𝜀4 Status-Dependent Longitudinal *CREB1* Genotype Effects on Individual Differences in Executive Function and Semantic Memory

**FIGURE 2:**
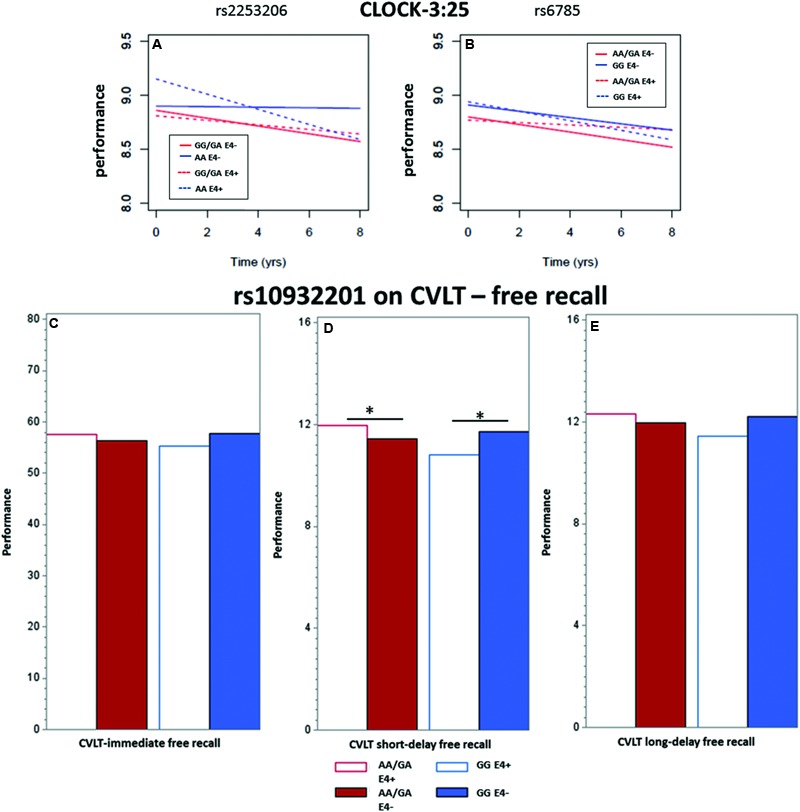
**Showing the significant interactions between *CREB1* SNP genotypes and APOE𝜀4 status on the longitudinal population-average trajectories for executive function and memory performance (A,B)** CLOCK-3:25 based on the predicted values after adjusting for baseline age, sex and education, and on population-average cross-sectional memory performance **(C)** immediate recall, **(D)** short-term and **(E)** long-term episodic memory based on the predicted values after adjusting for baseline age, sex, and education. All the effects were estimated using LME models except for CLOCK measures where GEE models were used. ^∗^ indicates significance at *p* = 0.05.

We observed a significant interaction (*p* < 0.01) between rs2253206 genotype and APOE𝜀4 status for the decline in executive function and semantic memory performance, measured by CLOCK-3:25 (**Table 2** and **Figure 2A**). Following-up this overall interaction effect with *post hoc* tests (Supplementary Table S16), we found significantly greater longitudinal decline in participants with the genotype AA and APOE𝜀4 positive compared to negative status. There were no differences in the longitudinal declines for the GG/GA genotype group by APOE𝜀4 status. For the same measure of executive function and semantic memory, we also observed that longitudinal decline in performance was significantly (*p* < 0.05) affected by the interaction between SNP rs6785 and APOE𝜀4 status (**Table 2** and **Figure 2B**). According to the *post hoc* tests (Supplementary Table S16), the effect of APOE𝜀4 status on longitudinal decline in performance was not significant in participants with either GG or AA/GA genotype.

**Table 2:** Interactions between interval, *CREB1* genotypes and APOE𝜀4 status significantly influence decline in executive function and semantic memory controlling for baseline age, sex, and education.

SNP and grouping	Cognitive measure	Estimate/s, *p*-value, Cohen’s d SNP main effect	Estimate/s, p-value, Cohen’s d SNPx interval interaction	Estimate, *p*-value, Cohen’s d APOE𝜀4 main effect	Estimate, *p*-value, Cohen’s d APOE𝜀4x interval interaction	Estimate/s, *p*-value, Cohen’s d SNPx APOE𝜀4 interaction	Estimate/s, *p*-value, Cohen’s d interval SNPx APOE𝜀4 interaction
**rs2253206 GG/GA vs AA**	CLOCK-3:25 transformed	0.131	0.003	-0.223	**0.054**	0.263	-**0.064**
		0.109	0.716	0.241	**0.003**^∗∗^	0.194	**0.001^∗∗^**
		0.20	0.22	-0.34	**1.19**	0.41	-**1.45**
**rs6785 AA/GA vs GG**	CLOCK-3:25 transformed	**0.111**	-0.007	-0.029	0.012	0.052	-**0.028**
		**0.079**	0.275	0.969	0.787	0.717	**0.045^∗^**
		**0.22**	-0.20	-0.04	0.29	0.09	-**0.76**

*Significant effects are shown in bold with ^∗^ indicating significance at *p* = 0.05 and ^∗∗^ at *p =* 0.01. The reference genotype group is underlined.*

### APOE𝜀4 Status-Dependent Cross-Sectional *CREB1* Genotype Effects of SNP rs10932201 on Individual Differences in Episodic Memory

The interaction between the SNP rs10932201 genotype and APOE𝜀4 status significantly affected the performance level of immediate recall, long-delay (both *p* < 0.05) and short-delay (*p* < 0.01) episodic memory (**Table 3** and **Figures 2C–E**). *Post hoc* testing (Supplementary Table S16) revealed that the level of performance was significantly lower in genotype group GG comparing APOE𝜀4 positive with negative status for short-term episodic memory (*p* < 0.05) and there was a tendency for similar effects for immediate and long-delay recall. In the other genotype group AA/GA, APOE𝜀4 positive compared to negative status was associated with significantly (*p* < 0.05) higher performance level for short-term episodic memory. No significant effects of APOE𝜀4 status were seen on performance levels for either immediate or long-term episodic memory in this genotype group.

**Table 3:** Interactions between *CREB1* genotype and APOE𝜀4 status significantly influence episodic memory controlling for baseline age, sex, and education.

SNP and grouping	Cognitive measure	Estimate/s, *p*-value, Cohen’s d SNP main effect	Estimate/s, *p*-value, Cohen’s d SNPx interval interaction	Estimate, *p*-value, Cohen’s d APOE𝜀4 main effect	Estimate, *p*-value, Cohen’s d APOE𝜀4x interval interaction	Estimate/s, *p*-value, Cohen’s d SNPx APOE𝜀4 interaction	Estimate/s, *p*-value, Cohen’s d interval SNPx APOE𝜀4 interaction
**rs10932201 AA/GA vs GG**	CVLT-immediate free recall	-0.145	-0.057	-2.486	0.010	**3.564**	-0.214
		0.83	0.438	0.388	0.251	**0.029**^∗^	0.201
		-0.02	-0.11	-0.27	0.02	**0.38**	-0.35
	CVLT-short-delay free recall	0.194	-0.017	-0.899	0.004	**1.428**	-0.070
		0.365	0.392	0.453	0.168	**0.004**^∗∗^	0.113
		0.07	-0.14	-0.34	0.03	**0.54**	-0.49
	CVLT-long-delay free recall	0.133	-0.023	-0.753	-0.014	**1.094**	-0.025
		0.522	0.198	0.389	0.204	**0.022**^∗^	0.555
		0.05	-0.14	-0.29	0.07	**0.43**	-0.21

*The reference genotype group is underlined. Significant effects are shown in bold with ^∗^ indicating significance at *p* = 0.05 and ^∗∗^ at *p* = 0.01.*

## Discussion

In this study, we examined whether three independent *CREB1* SNP genotypes contribute to individual differences in the performance level and longitudinal change of memory and executive function in older adults free of cognitive impairment. Consistently across our various measures the minor allele AA genotype for SNP rs2253206 and major allele GG genotype for SNP rs10932201 and rs6785 were associated with beneficial performance effects. The associations observed were significant adjusting for the already established risk factors such as age, sex, APOE𝜀4 status, and education. Our findings support a contribution of genetic variability in *CREB1* to individual differences in cognitive measures tapping primarily immediate recall and long-term episodic memory, semantic memory as well as both executive function and semantic memory.

In a recent genetic neuroimaging study, we found an association between the *CREB1* SNP rs10932201 genotype and individual differences in performance level and neural correlates of reward- and associative memory-based decision-making (Wolf et al., 2015). More specifically, the AA/GA compared to the GG genotype was associated with lower performance in memory and executive function as well as a decrease in task-related activation in, e.g., the hippocampus and cingulate gyrus. Here, we show increased longitudinal decline in performance of memory and executive function comparing AA/GA versus GG genotype groups. Accordingly, the common genotype (GG) for rs10932201 in Caucasians was consistently associated with beneficial effects on memory and executive function across our measures and studies. Importantly, allele frequencies were similar for this genotype in our American and European Caucasian samples (*A* = 0.46/0.48).

The minor allele A for SNP rs6785 has been associated with the risk for depression/bipolar disorder in Caucasians as well as smaller hippocampus volume and increased *CREB1* mRNA level in the prefrontal cortex of healthy Caucasian and African Americans (Li et al., 2014). Consistent with these previous observations, in our sample of healthy older Caucasian Americans, the A allele (AA/GA genotypes) for SNP rs6785 was associated with reduced immediate and long-term episodic memory performance.

We also found associations between the GG/GA versus AA genotype for SNP rs2253206 and lower performance level as well as increased longitudinal decline in performance of memory and executive function based on several cognitive measures (BNT, Category Fluency, CLOCKs, and CVLT-long-delay free recall). Previous research has linked this *CREB1* promoter SNP rs2253206 with differences in *BDNF* mRNA levels in the human hippocampus as well as with other SNPs in the genes encoding BDNF and G protein-activated K^+^ channel 2 (Juhasz et al., 2011; Lazary et al., 2011). Moreover increased rumination, thought rumination and current depression severity have been connected with the G major allele of rs2253206 (Juhasz et al., 2011; Lazary et al., 2011).

Our results also agree with a previous cross-sectional study in older, cognitively healthy adults, reporting associations of other *CREB1* SNPs, SNPs in the CREB1 binding protein gene (*CREBBP*), and another gene affecting the CREB1 pathway with episodic memory performance (Barral et al., 2014). Interestingly, these associations were, similarly to our findings, influenced by APOE𝜀4 status.

Consistently across those four measures affected by *CREB1*–*APOE*–interactions, the direction of cross-sectional and longitudinal effects of *CREB1* genotypes on performance was unchanged in combination with APOE𝜀4 negative but reversed with positive status. Reversal of the direction of genotype effect means the genotype formerly associated with better performance or less decline in performance changes to be associated with lower performance or more decline in performance. Respectively the genotype formerly associated with lower performance or more decline in performance changes to being associated with the opposite effects on performance level and decline. Such reversal of *CREB1* genotype effects on some measures of executive function, semantic and episodic memory in the presence of APOE𝜀4 positive status supports the idea of APOE𝜀4 positive status contributing to the deregulation of *CREB1* signaling.

Our results converge with previous findings in rodent models indicating the disturbance of CREB function in the presence of APOE𝜀4 genotype or protein. Specifically, it was shown that level of CREB activation as well as signaling up-stream and down-stream of CREB was decreased in cortex and hippocampus of transgenic mice with APOE𝜀4 positive compared to negative genotype status (Liu et al., 2015), and in rats injected with apoe4 compared to apoe2 protein (Qiao et al., 2014). Moreover these APOE𝜀4 genotype and apoe4 isoform-specific alterations of CREB signaling pathways were shown to enhance age-related decline in memory and impair *in vivo* hippocampal long-term potentiation.

The strengths of our analysis include comprehensive cognitive assessment over a mean follow-up period of 11 years, a mean sample size of 707 and a mean number of 4095 observations across 11 cognitive measures. In addition, our analysis incorporated the adjustment for already established risk factors including APOE𝜀4 status, sex, age, and education. As mentioned SNPs rs6785 and rs2253206 have been associated with depression and depression-related phenotypes (Juhasz et al., 2011; Lazary et al., 2011; Li et al., 2014), the prevalence of depression increases with aging (Arve et al., 1999; Stek et al., 2004) and there is a relation between depression and cognitive deficits (Lichtenberg et al., 1995; Trivedi and Greer, 2014). However, adding a measure of symptoms of depression (CESD) as a covariate did not change our findings. Our sample was homogenous regarding ethnicity, education and cognitive status. We found that the performance level and/or longitudinal decline in performance for the majority of the cognitive measures examined were affected by *CREB1* genotypes in adults during aging. Although significant effects were mostly of small size, the effect size for significant effects of these common polymorphisms on cognition varied ranging from Cohen’s *d* = -0.15 to -0.70. Small effects of SNPs are expected to be more frequent because of the multitude of genetic and environmental factors as well as their interactions that together contribute to individual differences in cognition (Wolf and Linden, 2012; Davies et al., 2015), cognitive aging and the risk of cognitive impairment.

Our current analyses were limited to a selection of only a few important factors that contribute to differences in human cognition during aging. Another concern is that we did not adjust for the number of SNPs and measures for executive function and memory tested. We argue that Bonferroni adjustment based on the number of analyses in our study is too conservative for the following reasons. (1) Our study was motivated by prior evidence for *CREB1* genotype effects on brain function and cognition based on different sample populations. (2) The direction of genotype effects is the same across samples and measures. (3) The direction of genotype effects is the same across baseline performance and longitudinal decline. (4) The measures were not independent because there is an overlap between the measure-based samples and measures were selected to target two cognitive phenotypes (memory and executive function), with some measures being more correlated than others, e.g., measures for both memory and executive function versus measures for memory only. (5) Even if we use the Bonferroni corrected significance level of 0.01 according to three independent SNPs and two independent phenotypes (memory and executive function) we find multiple significant effects of *CREB1* SNPs and *APOE4-CREB1* interactions on cognition. There are several sources that may limit the generalizability of our findings. First we restricted the analysis to only Caucasians and unimpaired visits, which left us for our largest sample with only 6104 observations because 206 visits were excluded due to the presence of dementia, 96 visits due to MCI, 5 visits due to impairment but not MCI, and 21 visits due to unknown type of impairment. Furthermore the BLSA is a sample with good health, high education and socioeconomic status levels on average.

One potentially important interaction partner for CREB appears to be BACE1, which besides cleaving APP may also regulate synaptic plasticity and myelination (Kandalepas and Vassar, 2014). Upregulation of BACE1 protein level has been reported to downregulate the cAMP-PKA-CREB signaling pathway independent of BACE1 activity for Aβ generation (Chen et al., 2012). CREB might influence BACE1 level because the human *BACE1* promoter contains a potential CREB binding site (Sambamurti et al., 2004). Genetic variants in CREB binding protein (CBP) have been associated with cognitive decline in human aging (Trompet et al., 2011; Barral et al., 2014). Other genes, besides being implicated in AD, are suspected to be linked with age-related cognitive decline in healthy aging, e.g., *APP* and *TOMM40* (Caselli et al., 2012; Jonsson et al., 2012; Davies et al., 2015; Payton et al., 2016) also may be regulated by CREB (Ge et al., 2004; Zhang et al., 2005; Tanis et al., 2008; Rodriguez-Tornos et al., 2013).

Importantly, CREB1 also mediates interactions between genetic and environmental factors by regulating the transcription of a large number of neuroplasticity-related genes dependent on neuronal activation during, e.g., social and cognitive activities (Wolf and Linden, 2012; Benito and Barco, 2015). Such experience-dependent regulation of CREB-signaling is involved in learning and memory formation. For example the learning experience-dependent activation of CREB by reelin has been shown to affect synaptic plasticity in the hippocampus (Brai et al., 2015), the regulation of synaptic plasticity genes and hippocampus-dependent associative learning (Telese et al., 2015).

We provide the first evidence for the influence of CREB on age-related longitudinal decline in memory and executive function in humans. The results from our previous (Wolf et al., 2015) and the current study suggest that *CREB1* gene variants contribute to individual differences in the level of memory and executive function performance in both young and older cognitively healthy adults. In addition to APOE𝜀4 status independent effects of *CREB1* genotypes, we also provide evidence for interactive effects between APOE𝜀4 status and *CREB1* genotypes on human cognition. The coexistence of APOE𝜀4 status dependent and independent effects of *CREB1* genotypes is supported by studies in animals showing APOE dependent and independent CREB signaling pathways.

In the future we will consider interactions between variants of *CREB1* and *CREB1* target genes or individual differences in environment-related factors such as cognitive and social activities.

## Author Contributions

CW designed the project, organized, analyzed and interpreted the genetic and behavioral data, and wrote the manuscript. YA, MB, and CG substantially contributed to the organization and statistical analysis of the data. TT carried out the genotyping. MK substantially contributed to the data collection. SR supervised the project. All authors critically revised and approved the manuscript.

## Conflict of Interest Statement

The authors declare that the research was conducted in the absence of any commercial or financial relationships that could be construed as a potential conflict of interest.
