# Supplementary material for: Cross-Sectional and Longitudinal Effects of CREB1 Genotypes on Individual Differences in Memory and Executive Function: Findings from the BLSA
Source: Front Aging Neurosci. 2017 May 16;9:142. doi: 10.3389/fnagi.2017.00142 (PMC5432543; doi:10.3389/fnagi.2017.00142)
Supplement: Supplementary file 1 [file Table_1.DOCX]

**Table S1.** Demographics of the Caucasian samples for all 11 cognitive measures based on tests of semantic and episodic memory (BNT, BVRT, CVLT), and both executive function and memory (CLOCKs, Fluency tests).

| Cognitive  performance measures | Sample  size | Number of observations | Mean | | Sex | | APOEε4  positive | Mean interval as mean time of follow-up (years) |
| --- | --- | --- | --- | --- | --- | --- | --- | --- |
|  |  |  | Baseline age  range | Years of education | W | M |  |  |
| BNT | 621 | 3381 | 68.60  39.2-93.0 | 16.49 | 270 | 351 | 151 | 8.54 |
| BVRT | 786 | 6104 | 49.61  17.7-92.0 | 16.44 | 365 | 421 | 199 | 21.85 |
| CLOCK-3:25 | 634 | 3026 | 68.74  39.2-93.0 | 16.46 | 276 | 358 | 156 | 8.41 |
| CLOCK-11:10 | 617 | 3299 | 68.53  39.2-93.0 | 16.48 | 268 | 349 | 150 | 8.57 |
| CVLT-immediate free recall | 733 | 4197 | 61.79  21.6-93.0 | 16.47 | 339 | 394 | 182 | 10.62 |
| CVLT-short-delay free recall | 733 | 4186 | 61.79  21.6-93.0 | 16.47 | 339 | 394 | 182 | 10.61 |
| CVLT-long-delay free recall | 733 | 4176 | 61.79  21.6-93.0 | 16.47 | 339 | 394 | 182 | 10.59 |
| CVLT-recognition discriminability | 733 | 4164 | 61.79  21.6-93.0 | 16.47 | 339 | 394 | 182 | 10.58 |
| CVLT-recognition response bias | 733 | 4164 | 61.79  21.6-93.0 | 16.47 | 339 | 394 | 182 | 10.58 |
| Category Fluency | 725 | 4170 | 64.79  29.2-93.0 | 16.47 | 321 | 404 | 188 | 9.60 |
| Letter Fluency | 725 | 4174 | 64.79  29.2-93.0 | 16.47 | 321 | 404 | 188 | 9.60 |
