# Supplementary material for: Cross-Sectional and Longitudinal Effects of CREB1 Genotypes on Individual Differences in Memory and Executive Function: Findings from the BLSA
Source: Front Aging Neurosci. 2017 May 16;9:142. doi: 10.3389/fnagi.2017.00142 (PMC5432543; doi:10.3389/fnagi.2017.00142)
Supplement: Supplementary file 2 [file Table_2.docx]

**Table S2.** Overview of cognitive measures and involved cognitive functions.

| Test | Cognitive measure | Memory component | Executive function component | Range | Involved cognitive functions |
| --- | --- | --- | --- | --- | --- |
| BNT | **Semantic memory and language**  BNT total correct | yes | no | 0-60  Our sample: 18-60 | Visual processing, semantic memory for objects and words, language production |
| BVRT | **Episodic immediate memory**  BVRT total errors | yes | no | 0-30  Observed: 0-23 | Visual episodic immediate memory for geometric figures, visuoconstruction |
| CVLT | **Episodic immediate memory**  CVLT immediate free recall total correct | yes | yes | 0-80  Observed: 5-80 | Verbal episodic immediate memory and executive function |
|  | **Episodic short-term memory**  CVLT short-delay free recall total correct | yes | yes | 0-16  Observed: 0-16 | Controlled retrieval from verbal episodic short-term memory in the presence of proactive interference with previous task |
|  | **Episodic long-term memory**  CVLT long-delay free recall total correct | yes | yes | 0-16  Observed: 0-16 | Verbal episodic long-term memory and executive function |
|  | **Episodic long-term recognition memory** CVLT discriminability  Success of discriminating targets from non-targets 🡪 higher values better | yes | no | Observed: -0.23-3.96 | Verbal episodic long-term recognition memory |
|  | **Episodic long-term recognition memory** CVLT response bias  Degree to accept an item when uncertain 🡪 values closer to zero better performance/less bias | yes | no | Observed:-0.98-1.98 | Verbal episodic long-term recognition memory |
| CLOCKs | **Executive function semantic memory and visuoconstruction**  CLOCK-3:25 total correct | yes | yes | 0-10  Observed: 3-10 | Executive function, semantic long-term memory and  visuoconstruction |
|  | **Executive function semantic memory and visuoconstruction**  CLOCK-11:10 total correct | yes | yes | 0-10  Observed: 4-10 |  |
| Fluencies | **Executive function, semantic memory and language**  Category Fluency total correct | yes | yes | 0 to infinity  Observed: 4.7-35.0 | Fluent language production, verbal semantic memory, executive function |
|  | **Executive function, lexical memory and language**  Letter Fluency total correct | yes | yes | 0 to infinity  Observed: 1.7-28.7 | Fluent language production, executive function, lexical memory, language processing |
