# Supplementary material for: Cross-Sectional and Longitudinal Effects of CREB1 Genotypes on Individual Differences in Memory and Executive Function: Findings from the BLSA
Source: Front Aging Neurosci. 2017 May 16;9:142. doi: 10.3389/fnagi.2017.00142 (PMC5432543; doi:10.3389/fnagi.2017.00142)
Supplement: Supplementary file 3 [file Table_3.DOCX]

**Table S3a.** SNPs within the *CREB1* gene with minor allele frequency (MAF) < .05 in

the Caucasian sample (N_max_ = 786) were excluded from further analysis.

Major allele in Caucasians marked in bold and risk-associated allele marked in red.

| CREB1 SNP | BP-units | MAF in our sample | Base change | Region |
| --- | --- | --- | --- | --- |
| rs2253206 | 208391978 | A = 0.45 | A/**G** | promoter |
| rs10932201 | 208426257 | A = 0.46 | A/**G** | intron |
| rs16839883 | 208429342 | G = 0.01 | **A**/G | intron |
| rs2254137 | 208444028 | C = 0.33 | **A**/C | intron |
| rs2709393 | 208454151 | A = 0.02 | **G**/A | intron |
| rs2551928 | 208465778 | A = 0.20 | A/**G** | 3'UTR |
| rs1045780 | 208467150 | A = 0.20 | A/**G** | 3'UTR |
| rs6785 | 208467997 | A = 0.20 | A/**G** | 3'UTR |

**Table S3b.** Pairwise LD analysis (R^2^) between the six SNPs with MAF > .05 and apoe4 in the Caucasian sample.

| CREB1 SNP | rs2253206 | rs10932201 | rs2254137 | rs2551928 | rs1045780 | rs6785 | APOEε4 |
| --- | --- | --- | --- | --- | --- | --- | --- |
| rs2253206 |  |  |  |  |  |  |  |
| rs10932201 | 0.1 |  |  |  |  |  |  |
| rs2254137 | 0.6 | 0.4 |  |  |  |  |  |
| rs2551928 | 0.2 | 0.2 | 0.1 |  |  |  |  |
| rs1045780 | 0.2 | 0.2 | 0.1 | 1.0 |  |  |  |
| rs6785 | 0.2 | 0.2 | 0.1 | 1.0 | 1.0 |  |  |
| APOEε4 | 0.0 | 0.0 | 0.0 | 0.0 | 0.0 | 0.0 |  |

**Table S3c**. All genotypes were distributed

according to Hardy–Weinberg equilibrium.

| SNP | HWE Chi-square | p-value |
| --- | --- | --- |
| rs2253206 | 0.598 | .439 |
| rs10932201 | 0.000 | .988 |
| rs6785 | 0.886 | .347 |

**Table S3d.** Demographics of genotype groups in the Caucasian sample (N=786). Genotype groups for all three SNPs were not significantly different for sex, baseline age, education or depression. Displayed are the numbers of subjects and the expected numbers (in brackets) in each group and Chi-square significance values for the categorical variables sex, and group means, standard deviations and p-value from ANOVA (2-tailed) for baseline age, years of education and depression.

| ***CREB1* SNP** | **Confounding factor** | | **Genotype group 0** | **Genotype group 1** | **Genotype group 2** | **p-Values (2-tailed, exact) for Pearson Chi-Square (DF = 1) / t-Test (DF = 87)** |
| --- | --- | --- | --- | --- | --- | --- |
| **rs2253206** | Sex | men | 132 (131.2) | 200 (202.5) | 89 (87.3) | .933 |
|  |  | women | 113 (113.8) | 178 (175.5) | 74(75.7) |  |
|  | Baseline age | | *M* = 50.64  *SD* = 17.22 | *M* = 49.20  *SD* = 16.14 | *M* = 48.99  *SD* = 17.71 | .506 |
|  | Education | | *M* = 16.43  *SD* = 2.10 | *M* = 16.37  *SD* = 2.29 | *M* = 16.60  *SD* = 2.15 | .526 |
|  | Depression score CESD | | *M* = 6.52  *SD* = 6.91 | *M* = 7.00  *SD* = 6.87 | *M* = 6.18  *SD* = 5.28 | .390 |
| **rs10932201** | Sex | men | 91 (90.0) | 212 (209.4) | 118 (121.6) | .854 |
|  |  | women | 77 (78.0) | 179 (181.6) | 109 (105.4) |  |
|  | Baseline age | | *M* = 50.34  *SD* = 16.27 | *M* = 48.94  *SD* = 16.75 | *M* = 50.21  *SD* = 17.32 | .541 |
|  | Education | | *M* = 16.32  *SD* = 2.22 | *M* = 16.49  *SD* = 2.14 | *M* = 16.43  *SD* = 2.29 | .703 |
|  | Depression score CESD | | *M* = 7.04  *SD* = 6.99 | *M* = 6.48  *SD* = 6.46 | *M* = 6.76  *SD* = 6.49 | .654 |
| **rs6785** | Sex | men | 19 (19.3) | 125 (130.7) | 277 (271.0) | .667 |
|  |  | women | 17 (16.7) | 119 (113.3) | 229 (235.0) |  |
|  | Baseline age | | *M* = 49.09  *SD* = 15.57 | *M* = 50.02  *SD* = 17.29 | *M* = 49.44  *SD* = 16.69 | .892 |
|  | Education | | *M* = 16.08  *SD* = 2.30 | *M* = 16.53  *SD* = 2.14 | *M* = 16.42  *SD* = 2.22 | .486 |
|  | Depression score CESD | | *M* = 8.91  *SD* = 11.08 | *M* = 6.31  *SD* = 6.70 | *M* = 6.71  *SD* = 6.07 | .103 |
