# Supplementary material for: Cross-Sectional and Longitudinal Effects of CREB1 Genotypes on Individual Differences in Memory and Executive Function: Findings from the BLSA
Source: Front Aging Neurosci. 2017 May 16;9:142. doi: 10.3389/fnagi.2017.00142 (PMC5432543; doi:10.3389/fnagi.2017.00142)
Supplement: Supplementary file 4 [file Table_4.docx]

**Table S4.** P-values and estimates in brackets for main effects and interactions of baseline age and interval obtained from separate models testing each of the three SNPs for effects on each of the 11 cognitive measures.

| SNP | rs10932201 (AA vs GA vs GG) | | | rs2253206 (GG vs GA vs AA) | | | rs6785 (AA vs GA vs GG) | | |
| --- | --- | --- | --- | --- | --- | --- | --- | --- | --- |
| Cog.  measure | AGE_0 | Interval | Interval  x age_0 | AGE_0 | Interval | Interval  x age_0 | AGE_0 | Interval | Interval  x APOE4 |
| CLK325  trans | **.003****  **(0.011)** | **.000*****  **(0.015)** | .064  (0.001) | **.004****  **(0.011)** | **.000*****  **(0.020)** | **.039***  **(0.001)** | **.003****  **(0.011)** | **.000*****  **(0.025)** | .743  (0.002) |
| CLK1110  trans | **.000*****  **(0.024)** | **.000*****  **(0.043)** | .210  (0.001) | **.000*****  **(0.023)** | **.000*****  **(0.040)** | .135  (0.001) | **.000*****  **(0.023)** | **.000*****  **(0.049)** | .209  (-0.012) |
| FLUCat | **.000*****  **(-0.112)** | **.000*****  **(-0.121)** | **.000*****  **(-0.013)** | **.000*****  **(-0.113)** | **.000*****  **(-0.102)** | **.000*****  **(-0.013)** | **.000*****  **(-0.112)** | **.000*****  **(-0.135)** | **.001****  **(-0.067)** |
| FLUlet | .618  (-0.008) | **.000*****  **(-0.058)** | **.000*****  **(-0.006)** | .644  (-0.007) | **.000*****  **(-0.036)** | **.000*****  **(-0.006)** | .655  (-0.007) | **.000*****  **(-0.062)** | .608  (0.011) |
| BOSCor | **.000*****  **(-0.151)** | **.000*****  **(-0.041)** | **.000*****  **(-0.010)** | **.000*****  **(-0.151)** | **.000*****  **(-0.048)** | **.000*****  **(-0.010)** | **.000*****  **(-0.151)** | **.000*****  **(-0.100)** | **.042***  **(-0.048)** |
| BVRTot | **.000*****  **(0.086)** | **.000*****  **(0.146)** | **.000*****  **(0.006)** | **.000*****  **(0.086)** | **.000*****  **(0.138)** | **.000*****  **(0.006)** | **.000*****  **(0.086)** | **.000*****  **(0.151)** | .707  (0.005) |
| CVLtca | **.000*****  **(-0.315)** | **.000*****  **(-0.365)** | .459  (-0.002) | **.000*****  **(-0.314)** | **.000*****  **(-0.366)** | .480  (-0.002) | **.000*****  **(-0.315)** | **.000*****  **-0.394** | **.061**  **(-0.144)** |
| CVLfrl | **.000*****  **(-0.074)** | **.000*****  **(-0.056)** | **.000*****  **(-0.002)** | **.000*****  **(-0.075)** | .**000*****  **(-0.046)** | **.001****  **(-0.002)** | **.000*****  **(-0.076)** | **.000*****  **(-0.070)** | **.088**  **(0.032)** |
| CVLfrs | **.000*****  **(-0.084)** | **.000*****  **(-0.072)** | **.001****  **(-0.002)** | **.000*****  **(-0.084)** | **.000*****  **(-0.065)** | **.001****  **(-0.002)** | **.000*****  **(-0.084)** | **.000*****  **(-0.075)** | **.022***  **(-0.046)** |
| CVLdis | **.000*****  **(-0.013)** | **.001****  **(-0.004)** | .403  (-0.000) | **.000*****  **(-0.013)** | **.002****  **(-0.003)** | .426  (-0.001) | **.000*****  **(-0.013)** | **.016***  **(-0.005)** | .052  (-0.007) |
| CVLbias | .784  (0.000) | **.000*****  **(-0.008)** | .156  (-0.000) | .787  (0.0002) | **.000*****  **(-0.008)** | .130  (-0.000) | .794  (0.000) | **.000*****  **(-0.004)** | .127  (-0.003) |
