# Supplementary material for: Cross-Sectional and Longitudinal Effects of CREB1 Genotypes on Individual Differences in Memory and Executive Function: Findings from the BLSA
Source: Front Aging Neurosci. 2017 May 16;9:142. doi: 10.3389/fnagi.2017.00142 (PMC5432543; doi:10.3389/fnagi.2017.00142)
Supplement: Supplementary file 5 [file Table_5.DOCX]

CREB1 genotype effects on 11 cognitive measures in American Caucasians. Transformed scores were used for CLOCKs. Adjusted for depression score p-values are indicated in brackets if depression score showed a trend or sig. main effect (ME) on cognitive measure. Standard error (SE). Significant effects are shown in bold with * indicating significance at p .05 and ** at p .01.

| Dependent Variable: CLOCK-3:25 total correct transformed  Model: (Intercept), SNP, Sex_clk, apoe4, educ_years, age0clk, interval_clk, SNP * interval_clk, age0clk * interval_clk, apoe4 * interval_clk, Sex_clk * interval_clk | | | | | | |
| --- | --- | --- | --- | --- | --- | --- |
| SNP and grouping | Estimate | SE | p-value ME SNP | Estimate | SE | p-value interaction SNPxinterval |
| rs10932201 overall  AA vs GG  AA vs GA  GA vs GG | -0.036  -0.032  -0.068 | 0.082  0.076  0.069 | .613(.617)  .662  .675  .324 | 0.008  0.005  0.014 | 0.009  0.008  0.007 | .183(.250)  .348  .498  .065 |
| rs10932201  AA/GA vs GG | -0.058 | 0.064 | .367 | 0.012 | 0.007 | .082 |
| rs2253206 overall  GG vs AA  GG vs GA  GA vs AA | 0.209  -0.145  0.064 | 0.087  0.064  0.086 | **.018*(.049*)**  **.015***  **.022***  .457 | -0.006  0.016  0.010 | 0.011  0.007  0.010 | .053**(.046*)**  .569  **.016***  .334 |
| rs2253206  GG/GA vs AA | 0.121 | 0.081 | .134 | 0.004 | 0.010 | .687 |
| rs6785 overall  AA vs GG  AA vs GA  GA vs GG | -0.048  0.175  0.127 | 0.125  0.131  0.065 | .116 (.113)  .703  .182  .051 | -0.002  -0.003  -0.004 | 0.016  0.017  0.017 | .816 (.837)  .923  .871  .524 |
| rs6785  AA/GA vs GG | 0.106 | 0.062 | .087 | -0.004 | 0.007 | .536 |

**Table S5.**

**Table S6.**

| Dependent Variable: CLOCK-11:10 total correct transformed  Model: (Intercept), SNP, Sex_clk, apoe4, educ_years, age0clk, interval_clk, SNP * interval_clk, age0clk * interval_clk, apoe4 * interval_clk, Sex_clk * interval_clk | | | | | | |
| --- | --- | --- | --- | --- | --- | --- |
| SNP and grouping | Estimate | SE | p-value ME SNP | Estimate | SE | p-value interaction SNPxinterval |
| rs10932201 overall  AA vs GG  AA vs GA  GA vs GG | 0.215  -0.146  0.069 | 0.148  0.120  0.131 | .300  .144  .220  .599 | -0.002  0.009  0.007 | 0.013  0.011  0.011 | .644  .891  .405  .516 |
| rs10932201  AA/GA vs GG | 0.116 | 0.125 | .351 | 0.004 | 0.011 | .682 |
| rs2253206 overall  GG vs AA  GG vs GA  GA vs AA | 0.364  -0.182  0.182 | 0.147  0.107  0.149 | **.031***  **.013***  .089  .222 | -0.008  0.025  0.017 | 0.013  0.010  0.012 | **.029***  .529  **.010***  .147 |
| rs2253206  GG/GA vs AA | 0.252 | 0.138 | .068 | 0.008 | 0.011 | .476 |
| rs6785 overall  AA vs GG  AA vs GA  GA vs GG | -0.018  0.281  0.101 | 0.259  0.267  0.112 | .469  .488  .293  .363 | 0.012  -0.020  -0.008 | 0.031  0.032  0.010 | .627  .712  .538  .394 |
| rs6785  AA/GA vs GG | 0.068 | 0.107 | .524 | -0.006 | 0.010 | .543 |

**Table S7.**

| Dependent Variable: Category Fluency total correct  Model: (Intercept), SNP, age0flu, Sex_flu, apoe4, educ_years, interval_flu, SNP * interval_flu, age0flu * interval_flu, apoe4 * interval_flu, Sex_flu * interval_flu | | | | | | |
| --- | --- | --- | --- | --- | --- | --- |
| SNP and grouping | Estimate | SE | p-value ME SNP | Estimate | SE | p-value interaction SNPxinterval |
| rs10932201 overall  AA vs GG  AA vs GA  GA vs GG | -0.146  -0.201  0.055 | 0.305  0.277  0.253 | .769(.855)  .633  .469  .828 | -0.017  0.020  -0.037 | 0.024  0.022  0.020 | .173(.166)  .482  .364  .065 |
| rs10932201  AA/GA vs GG | -0.006 | 0.238 | .979 | -0.031 | 0.019 | .101 |
| rs2253206 overall  GG vs AA  GG vs GA  GA vs AA | -0.090  0.092  -0.183 | 0.306  0.247  0.284 | .803(.855)  .768  .709  .521 | -0.056  -0.010  -0.046 | 0.025  0.019  0.023 | .073(.050)  **.028***  .621  .050 |
| rs2253206  GG/GA vs AA | -0.146 | 0.267 | .583 | -0.050 | 0.022 | **.025*** |
| rs6785 overall  AA vs GG  AA vs GA  GA vs GG | -0.266  -0.466  0.199 | 0.521  0.539  0.235 | .569(.570)  .609  .388  .397 | -0.007  -0.016  -0.023 | 0.041  0.042  0.019 | .469(.418)  .855  .711  .219 |
| rs6785  AA/GA vs GG | 0.139 | 0.225 | .536 | -0.021 | 0.018 | .240 |

**Table S8.**

| Dependent Variable: Letter Fluency total correct  Model: (Intercept), SNP, age0flu, Sex_flu, apoe4, educ_years, interval_flu, SNP * interval_flu, age0flu * interval_flu, apoe4 * interval_flu, Sex_flu * interval_flu | | | | | | |
| --- | --- | --- | --- | --- | --- | --- |
| SNP and grouping | Estimate | SE | p-value ME SNP | Estimate | SE | p-value interaction SNPxinterval |
| rs10932201 overall  AA vs GG  AA vs GA  GA vs GG | -0.239  0.171  -0.411 | 0.400  0.364  0.331 | .464(.422)  .550  .638  .216 | -0.023  -0.019  -0.004 | 0.025  0.023  0.020 | .611(.564)  .352  .393  .851 |
| rs10932201  AA/GA vs GG | -0.359 | 0.311 | .250 | -0.010 | 0.019 | .614 |
| rs2253206 overall  GG vs AA  GG vs GA  GA vs AA | 0.101  -0.006  0.107 | 0.402  0.325  0.373 | .956(.912)  .801  .985  .774 | -0.025  0.016  -0.041 | 0.026  0.020  0.024 | .217(.231)  .333  .408  .085 |
| rs2253206  GG/GA vs AA | 0.105 | 0.350 | .764 | -0.035 | 0.023 | .124 |
| rs6785 overall  AA vs GG  AA vs GA  GA vs GG | -0.181  -0.507  0.326 | 0.685  0.709  0.309 | .523(.535)  .792  .474  .291 | 0.003  0.013  -0.009 | 0.041  0.043  0.019 | .881(.850)  .933  .770  .633 |
| rs6785  AA/GA vs GG | 0.261 | 0.295 | .376 | -0.007 | 0.018 | .680 |

**Table S9.**

| Dependent Variable: Boston total correct  Model: (Intercept), SNP, age0bos, Sex_bos, apoe4, educ_years, interval_bos, SNP * interval_bos, age0bos * interval_bos, apoe4 * interval_bos, Sex_bos * interval_bos | | | | | | |
| --- | --- | --- | --- | --- | --- | --- |
| SNP and grouping | Estimate | SE | p-value ME SNP | Estimate | SE | p-value interaction SNPxinterval |
| rs10932201 overall  AA vs GG  AA vs GA  GA vs GG | 0.191  -0.353  0.544 | 0.392  0.356  0.326 | .226(.268)  .672  .322  .096 | -0.069  0.003  -0.072 | 0.028  0.025  0.023 | **.004**(.008**)**  **.013***  .894  **.002**** |
| rs10932201  AA/GA vs GG | 0.434 | 0.307 | .158 | -0.071 | 0.021 | **.001**** |
| rs2253206 overall  GG vs AA  GG vs GA  GA vs AA | 0.173  0.001  0.172 | 0.400  0.321  0.368 | .884(.808)  .665  .997  .641 | -0.049  0.007  -0.056 | 0.029  0.023  0.027 | .112**(.027*)**  .098  .744  .**039*** |
| rs2253206  GG/GA vs AA | 0.172 | 0.346 | .617 | -0.054 | 0.026 | **.039*** |
| rs6785 overall  AA vs GG  AA vs GA  GA vs GG | -1.215  -1.16  -0.052 | 0.687  0.711  0.305 | .209(.276)  .077  .102  .866 | 0.097  0.084  0.012 | 0.047  0.048  0.022 | .114(.224)  **.039***  .081  .570 |
| rs6785  AA/GA vs GG | -0.196 | 0.292 | .502 | 0.023 | 0.021 | .255 |

**Table S10.**

| Dependent Variable: Benton total errors  Model: (Intercept), SNP, age0bvr, Sex_bvr, apoe4, educ_years, interval_bvr, SNP * interval_bvr, age0bvr * interval_bvr, apoe4 * interval_bvr, Sex_bvr * interval_bvr | | | | | | |
| --- | --- | --- | --- | --- | --- | --- |
| SNP and grouping | Estimate | SE | p-value ME SNP | Estimate | SE | p-value interaction SNPxinterval |
| rs10932201 overall  AA vs GG  AA vs GA  GA vs GG | 0.291  -0.094  0.386 | 0.246  0.224  0.202 | .159(.152)  .237  .673  .057 | 0.028  0.029  -0.001 | 0.015  0.014  0.012 | .089(.090)  .064  **.036***  .945 |
| rs10932201  AA/GA vs GG | 0.359 | 0.191 | .060 | 0.008 | 0.011 | .504 |
| rs2253206 overall  GG vs AA  GG vs GA  GA vs AA | 0.210  0.272  -0.062 | 0.246  0.199  0.228 | .385(.174)  .394  .172  .784 | 0.021  0.008  0.013 | 0.015  0.012  0.014 | .349(.860)  .148  .497  .330 |
| rs2253206  GG/GA vs AA | 0.045 | 0.214 | .834 | 0.016 | 0.013 | .199 |
| rs6785 overall  AA vs GG  AA vs GA  GA vs GG | -0.398  -0.599  0.201 | 0.420  0.434  0.189 | .306(.229)  .343  .168  .288 | -0.012  -0.013  0.001 | 0.024  0.025  0.012 | .879(.813)  .635  .614  .915 |
| rs6785  AA/GA vs GG | 0.125 | 0.181 | .488 | -0.001 | 0.011 | .960 |

**Table S11.**

| Dependent Variable: CVLT-immediate free recall total correct  Model: (Intercept), SNP, age0cvl, Sex_cvl, apoe4, educ_years, interval_cvl, SNP * interval_cvl, age0cvl * interval_cvl, apoe4 * interval_cvl, Sex_cvl * interval_cvl | | | | | | |
| --- | --- | --- | --- | --- | --- | --- |
| SNP and grouping | Estimate | SE | p-value ME SNP | Estimate | SE | p-value interaction SNPxinterval |
| rs10932201 overall  AA vs GG  AA vs GA  GA vs GG | 0.030  0.718  -0.689 | 0.894  0.811  0.737 | .538(.603)  .973  .376  .351 | -0.092  -0.077  -0.015 | 0.093  0.085  0.077 | .570(.559)  .321  .361  .844 |
| rs10932201  AA/GA vs GG | -0.470 | .694 | .499 | -0.039 | 0.072 | .591 |
| rs2253206 overall  GG vs AA  GG vs GA  GA vs AA | -1.874  -1.144  -0.730 | 0.892  0.723  0.825 | .091(.106)  **.036***  .114  .376 | -0.069  -0.059  -0.011 | 0.094  0.076  0.085 | .686(.715)  .460  .442  .902 |
| rs2253206  GG/GA vs AA | -1.179 | 0.776 | .129 | -0.033 | 0.080 | .685 |
| rs6785 overall  AA vs GG  AA vs GA  GA vs GG | -1.001  0.580  -1.578 | 1.564  1.615  0.681 | .066(.080)  .523  .719  **.021*** | -0.069  -0.084  0.013 | 0.162  0.168  0.071 | .886(.870)  .668  .618  .846 |
| rs6785  AA/GA vs GG | -1.512 | 0.653 | **.021*** | 0.003 | 0.069 | .960 |

**Table S12.**

| Dependent Variable: CVLT-long-term free recall total correct  Model: (Intercept), SNP, age0cvl, Sex_cvl, apoe4, educ_years, interval_cvl, SNP * interval_cvl, age0cvl * interval_cvl, apoe4 * interval_cvl, Sex_cvl * interval_cvl | | | | | | |
| --- | --- | --- | --- | --- | --- | --- |
| SNP and grouping | Estimate | SE | p-value ME SNP | Estimate | SE | p-value interaction SNPxinterval |
| rs10932201 overall  AA vs GG  AA vs GA  GA vs GG | 0.185  0.219  -0.034 | 0.173  0.237  0.262 | .646(.666)  .480  .357  .876 | -0.043  -0.031  -0.012 | 0.023  0.021  0.019 | .164(.166)  .063  .139  .530 |
| rs10932201  AA/GA vs GG | 0.033 | 0.203 | .872 | -0.021 | 0.018 | .232 |
| rs2253206 overall  GG vs AA  GG vs GA  GA vs AA | -0.349  -0.342  -0.007 | 0.262  0.212  0.242 | .225(.262)  .182  .106  .978 | -0.039  -0.013  -0.027 | 0.023  0.019  0.021 | .233(.251)  .091  .509  .205 |
| rs2253206  GG/GA vs AA | -0.141 | 0.228 | .536 | -0.031 | 0.020 | .115 |
| rs6785 overall  AA vs GG  AA vs GA  GA vs GG | -0.320  0.073  -0.393 | 0.458  0.473  0.200 | .132(.153)  .485  .877  **.049*** | 0.010  0.016  -0.006 | 0.041  0.042  0.018 | .907(.952)  .814  .711  .735 |
| rs6785  AA/GA vs GG | -0.384 | 0.191 | **.045*** | -0.004 | 0.017 | .809 |

**Table S13.**

| Dependent Variable: CVLT-short-term free recall total correct  Model: (Intercept), SNP, age0cvl, Sex_cvl, apoe4, educ_years, interval_cvl, SNP * interval_cvl, age0cvl * interval_cvl, apoe4 * interval_cvl, Sex_cvl * interval_cvl | | | | | | |
| --- | --- | --- | --- | --- | --- | --- |
| SNP and grouping | Estimate | SE | p-value ME SNP | Estimate | SE | p-value interaction SNPxinterval |
| rs10932201 overall  AA vs GG  AA vs GA  GA vs GG | 0.098  0.049  0.049 | 0.271  0.246  0.223 | .936(.989)  .718  .842  .828 | -0.019  -0.012  -0.007 | 0.025  0.022  0.020 | .748(.756)  .448  .602  .729 |
| rs10932201  AA/GA vs GG | 0.064 | 0.210 | .763 | -0.011 | 0.019 | .578 |
| rs2253206 overall  GG vs AA  GG vs GA  GA vs AA | -0.408  -0.312  -0.096 | 0.271  0.219  0.250 | .238(.222)  .132  .155  .700 | -0.030  -0.020  -0.010 | 0.025  0.020  0.023 | .444(.469)  .232  .322  .668 |
| rs2253206  GG/GA vs AA | -0.219 | 0.235 | .352 | -0.017 | 0.021 | .423 |
| rs6785 overall  AA vs GG  AA vs GA  GA vs GG | -0.406  -0.064  -0.341 | 0.475  0.490  0.207 | .211(.277)  .393  .896  .099 | 0.010  0.023  -0.013 | 0.044  0.045  0.019 | .745(.758)  .819  .608  .489 |
| rs6785  AA/GA vs GG | -0.348 | 0.198 | .080 | -0.010 | 0.018 | .568 |

**Table S14.**

| Dependent Variable: CVLT-recognition discriminability  Model: (Intercept), SNP, age0cvl, Sex_cvl, apoe4, educ_years, interval_cvl, SNP * interval_cvl, age0cvl * interval_cvl, apoe4 * interval_cvl, Sex_cvl * interval_cvl | | | | | | |
| --- | --- | --- | --- | --- | --- | --- |
| SNP and grouping | Estimate | SE | p-value ME SNP | Estimate | SE | p-value interaction SNPxinterval |
| rs10932201 overall  AA vs GG  AA vs GA  GA vs GG | 0.034  0.032  0.002 | 0.050  0.046  0.042 | .752(.773)  .507  .485  .969 | -0.002  -9.9E^-5^  -0.002 | 0.004  0.004  0.004 | .791(.715)  .586  .980  .524 |
| rs10932201  AA/GA vs GG | 0.011 | 0.039 | .771 | -0.002 | 0.003 | .492 |
| rs2253206 overall  GG vs AA  GA vs GA  GA vs AA | -0.030  -0.023  -0.007 | 0.051  0.041  0.047 | .803(.892)  .555  .581  .876 | -0.005  -0.002  -0.003 | 0.004  0.004  0.004 | .507(.435)  .245  .584  .430 |
| rs2253206  GG/GA vs AA | -0.016 | 0.044 | .712 | -0.004 | 0.004 | .304 |
| rs6785 overall  AA vs GG  AA vs GA  GA vs GG | 0.020  0.116  -0.096 | 0.089  0.091  0.038 | **.038*(.054)**  .825  .206  **.013*** | -0.004  -0.004  -0.0001 | 0.008  0.008  0.003 | .872(.810)  .601  .624  .967 |
| rs6785  AA/GA vs GG | -0.082 | 0.037 | **.026*** | -0.001 | 0.003 | .862 |

**Table S15.**

| Dependent Variable: CVLT-recognition response bias  Model: (Intercept), SNP, age0cvl, Sex_cvl, apoe4, educ_years, interval_cvl, SNP * interval_cvl, age0cvl * interval_cvl, apoe4 * interval_cvl, Sex_cvl * interval_cvl | | | | | | |
| --- | --- | --- | --- | --- | --- | --- |
| SNP and grouping | Estimate | SE | p-value ME SNP | Estimate | SE | p-value interaction SNPxinterval |
| rs10932201 overall  AA vs GG  AA vs GA  GA vs GG | -0.020  -0.029  0.009 | 0.022  0.020  0.018 | .336(.331)  .359  .140  .611 | 0.001  -0.0003  0.001 | 0.002  0.002  0.002 | .826(.726)  .724  .868  .534 |
| rs10932201  AA/GA vs GG | 0.0001 | 0.017 | .994 | 0.001 | 0.002 | .548 |
| rs2253206 overall  GG vs AA  GG vs GA  GA vs AA | 0.017  0.018  -0.001 | 0.022  0.018  0.020 | .558(.452)  .440  .299  .942 | -0.001  -0.003  0.003 | 0.002  0.002  0.002 | .105(.074)  .773  .051  .149 |
| rs2253206  GG/GA vs AA | 0.006 | 0.019 | .773 | 0.001 | 0.002 | .401 |
| rs6785 overall  AA vs GG  AA vs GA  GA vs GG | 0.013  -0.034  0.047 | 0.039  0.040  0.017 | **.021*(.026*)**  .743  .393  **.005**** | -0.003  0.0002  -0.003 | 0.004  0.004  0.002 | .182(.204)  .480  .967  .074 |
| rs6785  AA/GA vs GG | 0.042 | 0.016 | **.008**** | -0.003 | 0.002 | .064 |
