# Supplementary material for: Cross-Sectional and Longitudinal Effects of CREB1 Genotypes on Individual Differences in Memory and Executive Function: Findings from the BLSA
Source: Front Aging Neurosci. 2017 May 16;9:142. doi: 10.3389/fnagi.2017.00142 (PMC5432543; doi:10.3389/fnagi.2017.00142)
Supplement: Supplementary file 6 [file Table_6.DOCX]

**Table S16.** Results for post-hoc tests following up significant interactions between SNP and APOEε4 status or SNP, APOEε4 status and interval. Significant results are highlighted in bold with * indicating significance at p .05 and ** at p .01.

| SNP | Cognitive measure | | Comparing APOEε4 positive and negative in  CREB1 SNP genotype group | Estimate | SE | p-value |
| --- | --- | --- | --- | --- | --- | --- |
| rs2253206 | Longitudinal  change in performance | CLOCK-3:25 transformed | GG/GA | -0.010 | 0.007 | .153 |
|  |  |  | AA | 0.054 | 0.018 | **.003**** |
| rs6785 |  |  | AA/GA | -0.017 | 0.010 | .109 |
|  |  |  | GG | 0.012 | 0.010 | .225 |
| rs10932201 | Level of  performance | CVLT-  immediate  free recall | AA/GA | 1.078 | 0.858 | .209 |
|  |  |  | GG | -2.486 | 1.384 | **.073** |
|  |  | CVLT-short-delay  free recall | AA/GA | 0.527 | 0.259 | **.042*** |
|  |  |  | GG | -0.899 | 0.419 | **.032*** |
|  |  | CVLT- long-delay  free recall | AA/GA | 0.341 | 0.251 | .175 |
|  |  |  | GG | -0.753 | 0.406 | .064 |
